# What is the evidence behind cancer care reviews, a primary care cancer support tool? A scoping review

**DOI:** 10.1007/s11764-022-01251-7

**Published:** 2022-09-06

**Authors:** Dipesh P. Gopal, Tahania Ahmad, Nikolaos Efstathiou, Ping Guo, Stephanie J. C. Taylor

**Affiliations:** 1 Centre for Primary Care, Wolfson Institute of Population Health, Barts and the London School of Medicine and Dentistry, Queen Mary University of London, London, England; 2 School of Nursing and Midwifery, Institute of Clinical Sciences, University of Birmingham, Birmingham, UK

**Keywords:** Living with and beyond cancer, Cancer survivorship, Cancer care reviews, Cancer, Primary care, Scoping review

## Abstract

**Purpose:**

A “cancer care review” (CCR) is a conversation between a patient recently diagnosed with cancer and primary care practitioner soon after a diagnosis of cancer in the UK. This scoping review aimed to identify: methodology and validated outcome measures used to evaluate CCRs, the impact of CCRs on quality of life or symptoms, and the views of patients, their carers and healthcare professionals on CCRs.

**Methods:**

A scoping review was performed and five databases (MEDLINE, Embase, PsychINFO, Scopus, Web of Science, Google Scholar) were searched systematically from January 2000 to March 2022.

**Results:**

Of 4133 articles, ten met the inclusion criteria. These included surveys, qualitative research on stakeholders’ views and a small study evaluating group consultation CCRs. There were no studies on methodology to evaluate CCRs or the impact of CCRs on patient quality of life or symptoms. Some primary care professionals felt CCRs were a tick-box exercise, and that they had inadequate time to deliver care, compounded by inadequate primary-secondary care coordination and lack of expertise which was echoed by patients. Interviews with patients found few recalled CCRs and those that recalled CCRs did, did not find them particularly helpful. Partners of patients would welcome CCRs to raise personal health concerns and remain updated on patient care.

**Conclusions:**

Further studies should identify the role that stakeholders believe they should have in CCRs, improve care coordination between primary care and secondary care and how to support caregivers.

**Implications for Cancer Survivors:**

There is currently insufficient evidence to support the use of CCRs in general practice.

## Introduction

**Fig. 1:**
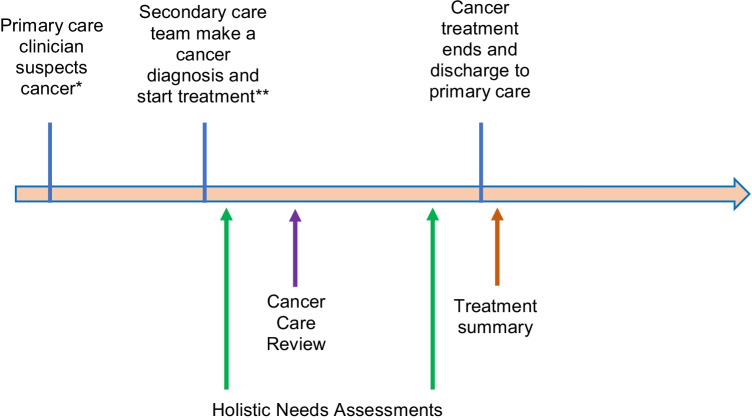
Schematic showing the timing of care plans alongside the timing of cancer treatment. Holistic needs assessments (HNAs) occur soon after a cancer diagnosis and close to the end of treatment often in secondary care. The cancer care review (CCR) is performed by a primary care clinician within 6 months of a diagnosis of cancer. 6 months is the time relevant to cited studies but since 2020 CCRs must be performed within 12 months of a diagnosis of cancer. A treatment summary is given at the end of treatment in secondary care when the patient is discharged to primary care. *The patient is referred by the GP to be seen by a secondary care clinician within 2 weeks. **Treatment must start within 2 months (62 days) from the date that the urgent referral is received from primary care

Cancer is the second leading cause of death, approximately accounting for one in six global deaths [1]. The burden of cancer is set to increase from 19.3 million cases in 2020 to 28.4 million cases in 2040 worldwide[2] with similar patterns seen in disability adjusted life years (DALYs) [3]. In the United Kingdom (UK), one in two people [4] born after 1960 will receive a diagnosis of cancer in their lifetime; however, nearly four out of ten will not die from it [5]. Whilst survival has doubled over the last 40 years [6], in the UK, the number of people who are living with and beyond cancer is set to increase from 2.5 million in 2015 to 4 million to 2030 [7]. Macmillan, the British cancer support charity, alongside NHS commissioners have introduced several initiatives to provide care for those who are living with and beyond cancer. These initiatives fall under the umbrella term of ‘personalised care for people living with cancer’ [8] and include the following: holistic needs assessments, cancer care reviews and treatment summaries (see Fig. 1). After diagnosis a one-off discussion with a primary care professional, such as a practice nurse or general practitioner (GP) should take place using a template called the cancer care review (CCR). After treatment has finished in secondary care, a summary of treatment is sent to primary care and the patient: treatment summary. Finally, there is support at health and well-being events in person pre-COVID, and online.

Within the UK, soon after a diagnosis of cancer, a person receives a holistic needs assessment (HNAs) [9] and a care plan through an oncology nurse in a secondary care setting. This helps identify patients’ concerns and signposts the patient to the appropriate supportive resources. Whilst HNAs are typically initiated in secondary care, there is primary care support using a cancer care review (CCR) [10]. Cancer care reviews were introduced in the UK in 2003[11]. These aim to help patients consolidate their understanding about their diagnosis, and signpost them to support including state benefits and preventative advice—such as smoking cessation. These are performed within 6–12 months after a diagnosis of cancer as a single intervention by a member of the primary care team, such as the general practitioner (GP) or practice nurse. Before 2020, CCRs were completed before 6 months after a cancer diagnosis, likely when many patients may be undergoing cancer treatment. However, recent 2021/2022 guidance suggests that CCRs can be completed up to 12 months after a cancer diagnosis, after which many patients may have completed cancer treatment [12].

In recent years, there have been review articles evaluating similar care assessments and plans such as holistic needs assessments [13], treatment summaries [14] and survivorship care plans [15]. A systematic review of HNAs suggested they had mixed impacts on patient outcomes such as mood and fatigue [13]. The authors concluded that the way the HNA is implemented along with the downstream care is more important that what is implemented. Treatment summaries, evaluated from mostly cross-sectional studies, were associated with greater patient understanding and better perceived care quality [14]. However, there were no studies evaluating the impact on patient outcomes. A meta-analysis of randomised controlled trials of survivorship care plans found that whilst they were acceptable to patients there was no detectable impact on patient reported outcomes [15]. Despite evaluation of care plans and assessments globally but there has been no formal evaluation of CCRs. A scoping review methodology was chosen over a systematic review methodology given the heterogeneity in CCR research to identify the breadth of literature on cancer care reviews [16]. For adults who are living with and beyond cancer, this scoping review aimed to answer the following research questions:

1. What methodology and validated outcomes has been used to evaluate cancer care reviews?
2. What is the evidence that cancer care reviews improve quality of life and patient symptoms?
3. What are the views of patients, their carers and healthcare professionals on cancer care reviews?

## Methods

A scoping review protocol was produced and reviewed by the team. The protocol was published online: https://osf.io/xrpbt/. The methodology of this scoping review is based on work by Arksey and O’Malley [17] and the more recent JBI guidance [18] which is based on Tricco and colleagues [19] and existing PRISMA guideline for scoping reviews [20]. The methodology includes identifying the research question, identifying relevant studies, study selection, charting the data collating, summarizing and reporting the results [21].

The population, concept of the research topic and the context such as geography or research type were defined (study selection step) prior to the definition of research questions, according to scoping review guidance [19, 22].

The *population* is adults who are living with and beyond cancer, a term which is generally synonymous with cancer survivor which is defined as anyone with a diagnosis as cancer regardless of their place within a disease course [23]. The *concept* is cancer care reviews. The *context* is English language primary and secondary quantitative and qualitative research in primary and secondary healthcare settings, as well as reports, analysis or discussion articles and letters. Conference abstracts were excluded. Searches were limited to English language since CCRs originated in the UK.

**Fig. 2:**
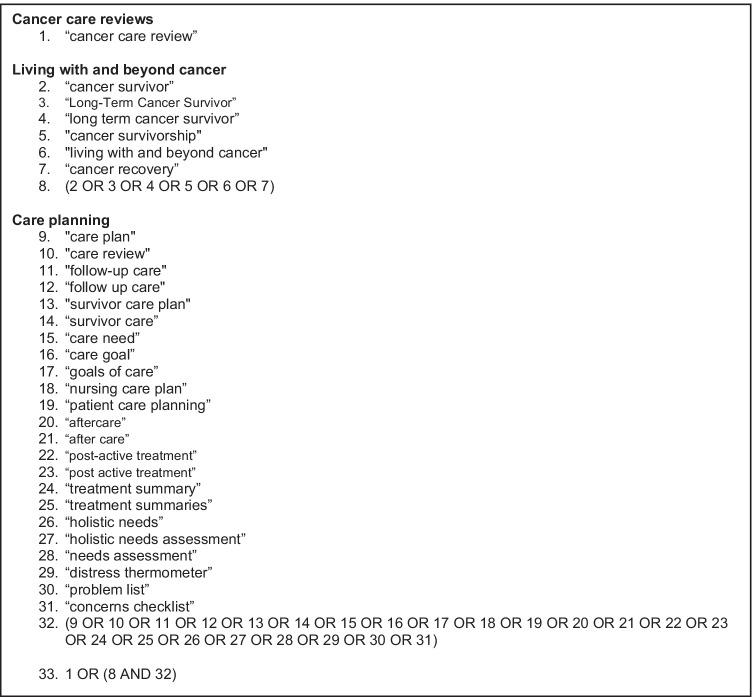
Search strategy for this scoping review

MEDLINE, SCOPUS, PsychINFO, EMBASE, Web of Science and Google Scholar were searched using the search strategy shown in Fig. 2 from January 1, 2000, until March 3, 2022. The year 2000 was chosen as a cut-off, since cancer care reviews were introduced into the UK in 2003 [11]. Forward citation, meaning looking at the papers that had cited the existing paper, and backward citation, meaning looking at the references cited within a paper, were used to ensure inclusion of all relevant studies.

All studies were downloaded as Endnote files from their respective databases and duplicate records were removed. The remaining records were uploaded to an online systematic review platform ‘Rayyan’ [24] and records were independently screened (DPG, TA) to identify eligible studies based on inclusion criteria. Any conflicts were resolved through consensus between screening researchers and an independent researcher (SJCT) if required. Full text of the remaining records was independently screened (DPG, TA) and data was extracted from the studies fulfilling inclusion criteria. The same procedures for conflict resolution as in title and abstract screening were followed for full text screening.

### Data extraction

The results were extracted by a single researcher (DPG) into a data chart which was developed iteratively into the following categories: lead author name, year of publication, year of data collection, study location, study design, type of subjects involved, sample size, mean age, % female, % minority ethnic, % disability, featured cancer types, study findings, study limitations, CCR relevant findings. Data extraction was independently verified by another researcher (SJCT) and there was 100% agreement. Data charting could not be calibrated due to novel study methodology and study heterogeneity. Quality appraisal and/or risk of bias assessments were not conducted as they are deemed unnecessary for scoping reviews [18]. The data was synthesised using the patterns, advances, gaps, evidence for practice and research recommendations framework [25].

## Results

**Fig. 3:**
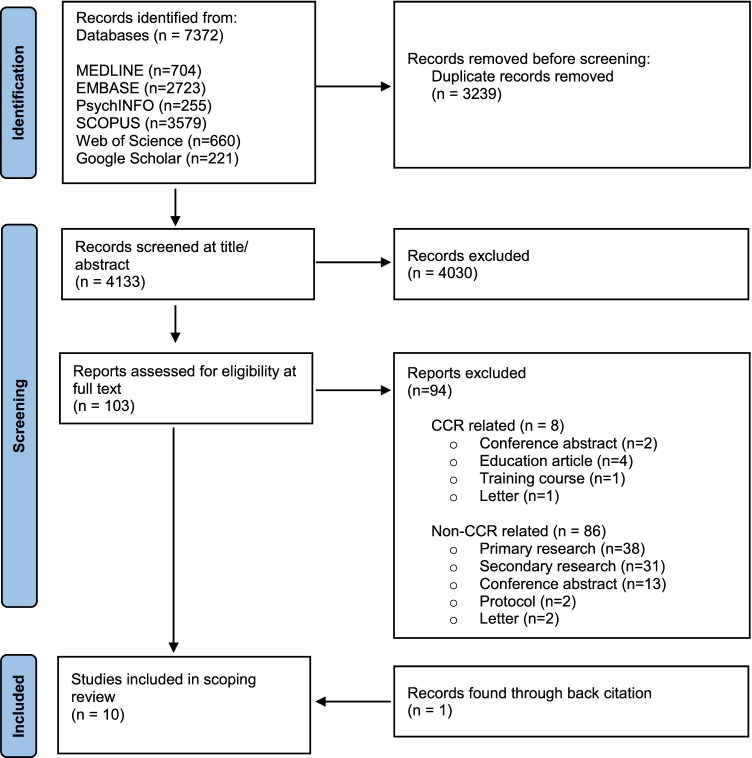
PRISMA flow diagram showing records included through systematic search of the literature

Database searches retrieved 7372 records and 4133 records remained after exclusion of duplicates. Following screening by title and abstract, inclusion and exclusion criteria were applied to 103 full text articles. Of these articles, ten articles [11, 26–34] fulfilled inclusion criteria, of which one was identified through back citation (see Fig. 3).

There were five qualitative studies [11, 27, 30, 31, 33], four surveys [26, 28, 32, 34] and one letter to the editor [29] which identified views of CCRs from patients (*n* = 3) [11, 29, 30, 33], their partners who were sometimes also carers of patients (*n* = 1) [27], and healthcare professionals(*n* = 5) [11, 26, 28, 29, 31–33], including primary care professionals (see Table 1). There was one study measuring the impact of CCRs performed via group consultations [34]. There were no studies identifying validated outcome measures to evaluate CCRs, the impact of CCRs on quality of life (patients or their carers or spouses) or patients’ symptoms.

**Table 1:** Summary table of included studies in scoping review on cancer care reviews (CCRs). These included online surveys, interviews and focus groups. *n/a*, not applicable. None of the papers reported disability. *GPs*, general practitioners

Author, year of publication	Year of data collection	Location	Study design	Data collection	Subjects						Key findings relevant to CCRs
					Type	Sample size	Mean age (± SD)	Female (%)	Ethnic minority (%)	Cancer types	
Adams et al. 2011 [11]	2009–2010	Thames Valley, England; UK	Qualitative	Focus groups – face-to-face	Primary care healthcare professionals	71 (31 GPs, 1 GP trainee, 1 medical student, 13 GP nurses, 2 district nurses, 6 practice managers, 1 phlebotomist, 1 healthcare assistant, 15 admin	Professionals: unknown	Professionals: unknown	Professionals: unknown	Various—breast, prostate, colorectal, head and neck, lung, melanoma, testicular, endometrial, uterine, pelvic, Hodgkin’s, non-Hodgkin’s, bladder, renal	Practice staff were unsure of the exact role of primary care in long-term cancer care: some thought that primary care should provide a supportive role whilst others felt that that their role should only be during the palliative phase. Patients noted that primary care staff failed to acknowledge their cancer diagnosis: such an acknowledgement would have been beneficial. Some patients were unsure whether primary care had sufficient expertise for cancer care and felt that lack of coordination between primary and secondary care negatively impaired continuity of care.
				Interviews, face-to-face;	Patients	38	Patients mostly aged 50–70 (14/38) and > 70 (16/38)	Patients: 50%	Patients: 0%		
Adams et al. 2012 [27]	2009–2010	Thames Valley, England; UK	Qualitative	Interviews, face-to-face	Partners of patients	22	Mostly aged 50–70 (14/22)	68%	5%	Various—prostate, colorectal, head and neck, lung, melanoma, breast, Hodgkin’s lymphoma, testicular, gynaecological, bladder	Partners of patients encountered several challenges when caring for patients with cancer. One example was providing nursing care after hospital discharge/procedure without the appropriate expertise or experience. They encountered anxiety about providing future care and found the provision of emotional support stressful, negatively impacting their personal relationships. Partners felt unable to voice concerns about the impact of caring on their own health and well-being.
Kendall et al. 2013 [33]	Unknown	England, Scotland; UK	Qualitative	Interviews, face-to-face	Primary care healthcare professionals	29 (27 GPs, 2 GP nurses)	Unknown	Unknown	Unknown	Various—breast, prostate, colorectal, lung, haematological, bladder, skin	This feasibility study tested the implementation of a structured template for cancer care reviews called Cancer Ongoing Review Document (CORD). GPs and practice nurses felt that the CORD template needed to be better integrated into the electronic system. Some found the prompts helpful, but others thought it made consultations ‘tick list’. Few patients could remember the proactive invitation for the consultation, or a special document being filled out. Members of the lay advisory group noted that an explanation of the consultation or a printout of the completed document would be helpful. A review of the medical notes identified that CCRs were carried out 3 months after diagnosis and GPs carried out the majority of CCRs. Most CCRs were carried out in clinic (GPs 50%, GP nurses 80%) whilst the remainder of CCRs were carried out on the telephone and rarely during home visits.
					Patients	16	66	43%	unknown		
Williamson et al. 2020 [31]	2015	England, Scotland, Wales; UK	Qualitative	Interviews, telephone/face-to-face	Primary care and oncology healthcare professionals; managerial staff	21 (total)	Unknown	Unknown	Unknown	n/a	Professionals and clinicians thought that long-term cancer follow-up in secondary care was costly and unsustainable. They noted a lack of coordination between primary care and secondary care services and variable amounts of self-management support for patients. Interview participants identified the challenges of implementing the ‘Recovery Package’ and the need for better use of technology and evidence base for survivorship initiatives.
					- Primary care staff	10 (2 GPs, 5 oncology nurses, 3 oncologists)					
					- Managerial staff	8 (6 nurses, 2 managers)					
					- Clinical and managerial staff	3 (2 GPs/commissioners 1 nurse/commissioner)					
Beaver et al. 2020 [30]	2018	London/ Preston England; UK	Qualitative	Interviews, face-to-face	Patients	17	59.4 ± 10.8	100%	6%	Endometrial cancer	Patients found hospital follow-up helpful initially for reassurance but inconvenient when travelling, parking, time taken especially if there were no clinical problems apparent. Self-management was empowering with the right support such as looking out for symptoms of recurrence. Patients were often unable to recall certain aspects of care planning. Only 2/17 received a treatment summary; 6/17 remembered information on health and well-being events; all were unsure about the contents of a CCR.
Dilley and Can 2019 [29]	n/a	Chelmsford, England; UK	n/a	Letter to editor	Individual opinion of healthcare professional/patient	n/a	Unknown	n/a	n/a	n/a	As a GP and patient, the author believes that CCRs and their associated funding for their implementation could facilitate continuity of care for patients.
Watson et al. 2010 [26]	Unknown	England; UK	Quantitative	Online survey & audit of 65 oncology clinic letters	Primary care (not specified) and oncologists	300	Unknown	38%	Unknown	n/a	Most primary care professionals offered CCRs and conducted CCRs themselves. 40% of staff found CCRs helpful whilst 60% found CCRs helpful for patients. 67% of primary care staff were dissatisfied with communication with secondary care and dissatisfied about access, secondary care advice and appointments. Most oncologists communicated clear information given about treatment and follow-up but less often the long-term impact of cancer such as late effects or symptoms of recurrence which primary care staff felt was missing from communication. This finding was corroborated by the oncology discharge letter audit.
Walter et al. 2015 [28]	2014	UK	Quantitative	Online survey	Primary care healthcare professionals (all GPs)	500	Unknown	25%	Unknown	n/a	50% of GPs reported receiving a detailed cancer treatment summary, details of ongoing care from the hospital, or details of ongoing care to be provided by the GP. 16–21% of GPs received information on late effects from secondary care, and the appropriate time to refer to secondary care in case of recurrence. 76% of GPs felt cancer treatment summaries and survivorship care plans would improve their ability to provide care. As well as this, 77–81% of GPs believed that primary and secondary care should be jointly responsible for bone and cardiovascular health for people living with and beyond cancer. 50% of GPs reported having received previous training on the care of people living with and beyond cancer and most GPs were keen to learn more about providing cancer care.
Dyer and Dewhurst 2020 [32]	2018	Southwest London, England; UK	Quantitative	Online survey	Primary care healthcare professionals (all GP nurses)	123	Unknown	Unknown	Unknown	n/a	The survey of practice nurses showed 60% were clear about their role in supporting people with cancer, but 1 in 6 were confident about conducting CCRs. 43% of respondents were unsure or did not understand the role of CCRs, Practice nurses were more confident about discussing hormone treatment and surgery but less confident about immunotherapy. Respondents were more confident supporting patients with lifestyle advice as well as physical and psychological issues. They were less confident about discussing sexual dysfunction, and signs of recurrence. More than 70% of practice nurses had not received cancer specific training citing barriers such as lack of time and distance to training locations.
Hodgson 2020 [34]	Unknown	Lancaster, England; UK	Quantitative	Unblinded single arm trial	Patients	6	unknown	unknown	unknown	unknown	CCRs for 6 people living with and beyond cancer were conducted via 2 group consultations performed across 3 months. There was a decrease in physical, emotional, lifestyle and information needs by 58% before the first and after the second group consultation as measured by the number of needs through holistic needs assessments. Patients and clinicians were satisfied with this format of consultation, but this was not measured formally.

In surveys[26, 28] involving healthcare staff, 25–38% respondents were female whilst gender was not reported in an interview study[31], a focus group study[11], or a trial [34] involving healthcare professionals. Fifty percent of patients interviewed for one study were female[11] and another study only included participants who had endometrial cancer[30]. An interview study[27] involving partners of patients had 68% female participants. No studies reported data on disability and some studies[11, 26, 28, 31–34] did not report ethnicity. In those that did report this[27, 30], 5–6% of patients or partners were from ethnic minority groups.

### Patient views on cancer care reviews

This data was sourced from interviews with patients in GP practices with various cancer types within 6 years of diagnosis (*n* = 38) [11] and within 3 months of diagnosis (*n* = 16) [33], interviews with patients (*n* = 17) from gynaecology outpatient clinics diagnosed with stage I endometrial cancer within the last year [30], and a letter from a GP who had been diagnosed with cancer [29]. Adams and colleagues[11] conducted interviews with patients within three years of their cancer diagnosis, where just 5% (*n* = 2) recalled a CCR and more than half (*n* = 20) could not recall any detailed discussion with their primary care team. This was echoed in the study by Kendall et al. [33] where few patients could not remember either having a specific proactive cancer related appointment or a CCR. A more recent qualitative interview study [30] conducted with patients with stage I endometrial cancer indicated that some were unsure of what a CCR involved, and the difference compared to a routine GP appointment. Only those who reported the existence of a good relationship with a GP were positive about CCRs with some not keen to discuss their cancer diagnosis with unfamiliar practitioners in a supplementary appointment dedicated to CCRs: “Well the thing is, I don’t feel comfortable with my GP because the one I had is retired. And every time I go to phone up now, I get somebody different. And I've built no relationship up with them. They don’t know me, they don’t really know my condition.” (ID 11, page 6) [30].

A letter [29] written by a GP who had a cancer diagnosis suggested that CCRs and their associated funding is important to provide an access route to primary care: “QOF cancer care reviews can provide a valuable doorway allowing patients to access this support. As a patient, I vote that they should remain.”

Those patients that had CCRs (*n* = 2) [11] did not find them particularly helpful as primary care staff seemed unaware of the patient’s history and ongoing cancer treatment, which may be in part not reading clinical notes, and a general lack of awareness of novel cancer treatments:

“They invited us to go and see them as a follow-up, but she was not aware of the operation I’d actually had, and she was not aware what they had in fact done, and she, sitting discussing with her ‘they really do that now do they?’” (P20, 68-year-old male, pages e176-7) [11].

“He hadn’t looked at the notes, it was almost like I kind of went through ‘this is what I’ve got, these are the drugs I’m having’, which was … Trying to remember all those, so it wasn’t really, there was no value to me at all at that point frankly, it was a bit of a waste of time quite honestly.” (P16, 36-year-old male, page e177) [11].

Patients were unsure about the right time for a CCR with some preferring a review with primary care soon after diagnosis and others preferring contact at the end of treatment or several reviews during cancer treatment. A proactive approach to cancer care via a CCR would legitimise concerns to seek help in primary care and patients mentioned several things that should be included in primary care cancer-specific reviews, but which are not formally part of CCRs. These included the following: illness acknowledgement, an explanation of cancer in lay terms, support for children, emotional and psychological support for themselves and, information about the symptoms of recurrence, travel insurance, and local support groups.

Patients recognised that barriers to providing cancer care, which may include CCRs, in GP practices may arise from lack of coordination between primary care and secondary care, with some secondary care professionals denigrating the value of GPs in cancer care: “I fear that there’s no consistency between specialists and GPs, because I like to feel as though I trust my GP, but when they start bickering about ‘oh your GP doesn’t know anything’, you can be easily swayed, and you say, ‘should I be talking to my GP about this?’.” (37-year-old male, page e178) [11].

Others recognised lack of time as a potential barrier to providing a CCR within a 10-min consultation which stopped patients raising concerns with GPs or practice nurses about their cancer:

“Some sort of follow-up thing would be nice because there are things you’d like to ask because when you do come back here for your check-up they’re so pushed for time, you haven’t, they obviously haven’t really got a lot of time.” (P52, 74-year-old male, page e178) [11].

### Partner views on cancer care reviews

Interviews with partners of patients (*n* = 22) who had been diagnosed with various types of cancer within the last 3 years were the only data source. Most partners of patients [27] (*n* = 17, 77%) who had various types of cancer were in favour of having their own cancer care review, designated review time, to discuss symptoms of recurrence for the patient: “I’d like maybe to talk about the likelihood of it coming back, or him developing a different kind of cancer, touch wood, that would put my mind at rest…” (P155, wife, separate interview, page 2791) [27].

Other partners thought that having a personal CCR appointment for themselves would allow them to stay up-to-date with their fellow partner who was undergoing cancer treatment: “Yes, that would be a good idea, yes, yes, it’s best to know what’s going on, I mean if you have it first hand you know the governor is not giving you a load of cobblers to keep you quiet, don’t you, no, I think that would be a very good idea” (P1214, husband, separate interview, page 2791) [27].

A dedicated appointment for a partner would provide an opportunity to discuss their own concerns relating to their own health but specifically being a carer: “We’re coping OK, but given all the things I’ve read about carers, getting stressed and all that kind of thing, it does surprise me that now this has been happening for 18 months, nobody has ever suggested that I should just have a check up, or talking to me to see if I’m caring [sic] …” (P174, wife, separate interview, page 2791) [27].

Partners of patients with cancer found that primary care tended to be for patients rather than for carers. This acted as a perceived barrier to seeking help about their own health or caring needs. Others found that the confidentiality of the doctor-patient relationship limited disclosure of information relevant to their caring responsibility, such as when discussing patient care at a dedicated appointment for the partner. Furthermore, partners thought that primary care professionals lacked knowledge of family roles, such as knowing the partner or main carer.

### Primary care professional views on cancer care reviews

Professional views were derived from a variety of sources: a 2010 survey of 100 oncologists and 200 primary healthcare professionals with an audit of oncology discharge letters [26], a 2015 survey of 500 GPs [28], a 2020 survey of 123 GP practice nurses [32], a 2011 focus group of 6 GP multidisciplinary teams [11], a 2013 interview study of 29 primary healthcare professionals [33], and a 2020 interview study with 19 primary and secondary care clinical and managerial staff[31].

#### Understanding of CCRs

Only one survey checked understanding of the concept of CCRs and found that of those practice nurses who were clear in supporting patients with cancer, 43% (*n* = 32) did not correctly understand or were unsure about the purpose of CCRs [32].

#### How CCRs are carried out

Survey data of GP practices and primary care staff identified that most CCRs were performed by GPs. In a survey of 200 GPs [26], 98% performed CCRs themselves but some practices reported delegating this to practice nurses (14%), district nurses (19%), palliative care nurses and community matrons (6%). In the same survey, just over half the GPs (51%) conducted CCRs opportunistically during a consultation with a similar proportion (45%) reported by Walter and colleagues [28] in a survey of 500 GPs. This was similarly reflected in focus groups with primary care teams where CCRs were completed opportunistically in person or on the telephone [11]. There was no consensus on the optimum timing of CCRs and very few performed CCRs at a set time point. Only 17% were performing CCRs at a set time whilst 5% were performing regular reviews in the study by Watson [26]. In the same study, 39% aspired for CCRs at different times of the patient journey such as at diagnosis (62%), end of outpatient follow-up (53%) and six-monthly (60%).

Between 40 and 64% of GPs used a CCR template or structure [26, 28] with practice or local CCG templates being the most popular [28]. However, participants in focus groups [11] and interviews [33] had mixed views on the value of templates in CCRs with some GPs finding them a useful structure and others thinking CCRs were a “tick-box exercise”—thereby fundamentally changing the consultation structure:

“I don’t think it’s of any value personally, I don’t think it’s to the patient benefit at all, I think it’s just another hoop you have to jump through if you want to get paid.” (practice 5, GP partner, page e179) [11].

“I do have slight anxieties about making everything so structured, I mean the ethos has always been “we’re accessible, we’re here if you want us, if you do come in we don’t have to follow a template, we can go by your agenda, and what you’re worried about”, and I have concerns about templates.” (practice 2, GP partner, page e179) [11].

Some GPs and practice nurses found that filling the CCR documentation with the patient was mutually beneficial and improved clinical practice and documentation [33].

One GP [29] who had experience of having cancer treatment identified a potential driver for cancer care review as ticking of the financial incentive, Quality and Outcomes Framework (QOF) box. This was corroborated by two participants of a study by Williamson [31] including healthcare professionals, managers and commissioners interviewed, who suggested that financial incentives may support related initiatives such as holistic needs assessments which inform CCRs.

Walter and colleagues [28] found that 53% of GPs found CCRs useful whilst 10% did not specify in which regard. GPs who performed CCRs with a template and those who made specific appointments were more likely (48% and 327%, respectively) to find CCRs useful compared to those who did not use templates, or performed CCRs opportunistically. GPs with specific appointments for CCRs found CCRs three times more useful even after adjusting for template use compared to those that performed CCRs opportunistically. Watson [26] reported 40% of primary care staff found CCRs useful to staff and 60% found them useful to patients.

A 2010 [26] and 2015 [28] survey showed that most GPs (> 50%) discussed psychological symptoms and support during CCRs. However, Watson[26] in 2010, found that in addition most GPs reviewed treatment, patient follow-up and discussed family needs. Walter’s [28] more recent survey found that most GPs discussed treatment-related side effects and lifestyle. However, Watson [26] in 2010 found 68% of GPs discussed social support, such as work and finances, compared to 36% of GPs in 2015 [28]. In addition, 19% fewer GPs discussed lifestyle or healthy behaviours in 2010 [26] compared to 2015 [28]. The content of CCRs in Walter’s study [28] such as lifestyle advice or social support was significantly related to GP confidence in discussing these topics. Only a minority of GPs provided information on symptoms of recurrence, familial or genetic risks of cancer, and screening requirements in Watson’s study [26]. These topics were not included in the survey by Walter and colleagues [28]. A 2020 survey of practice nurses [32] found practice nurses were more confident in discussing hormone treatment and surgery but less confident in discussing immunotherapy. Furthermore, practice nurses were more confident in discussing physical and mental health problems as well as lifestyle advice such as smoking cessation. However, they were less confident at discussing long term effects such as sexual dysfunction or the effect on fertility alongside signs of recurrence and the need for follow-up testing.

#### Barriers to the implementation of CCRs

Implementation of CCRs, as part of the Recovery Package, by clinicians and policymakers was noted to be difficult at a time of ‘decreasing resources’[31]: “We’re already having talks with our commissioners which are being led by our local cancer network to look at how we can jiggle the funds around really and commission the Recovery Package activity, but that would be on the proviso obviously that we reduce the follow-up ‘cos there won’t be more money and we’ve got a lot more patients coming in …’” (ID 11 Lead Cancer Nurse, page 4) [31].

This allusion to a lack of time to conduct CCRs was reflected in Adams and colleagues’ paper [11]: “One of the things really struck me was the patients really wanted a lot of information, and to some extent I think the sort of cancer care review process is probably not the place for that, I mean I think a few good websites and information sheets or a few helplines for the patients, they could actually be more useful, I think you could do a lot of that outside of a GP setting, if there was more sort of an information infrastructure that would be helpful.” (practice 3, GP partner, page e180) [11].

Other barriers to implementation of CCRs by the primary care team included lack of knowledge in long-term cancer care [11]: “I also feel you know, I probably don’t know enough about the subject to give advice, but I think from an emotional point of view, yeah, you just sometimes you just have to listen, don’t you.” (practice 5, practice nurse, page e180) [11].

This was more evident on a survey [28] including a measure of GP self-reported knowledge with 13–30% more GPs appreciative of the association of cancer treatment with bone health compared to cardiovascular health depending on specific cancer treatment. GPs were almost 5 times (OR 4.76 [95% CI = 3.07 to 7.64], *P* < 0.001) more likely to consider a history of cancer when assessing bone health compared to cardiovascular health. Most GPs were keen to undertake training in treatment-related side effects and the long-term effects of cancer treatment. Over 77% of GPs felt that both primary and secondary care should jointly manage bone and cardiovascular health. Some lack of knowledge may be attributed to a lack of training which was evident in the survey of practice nurses by Dyer and Dewhurst [32] who found over 70% (*n* = 89) had not had cancer-specific training. Conversely, 15% (*n* = 18) had had cancer-specific training. Barriers to accessing such training for practice nurses included lack of training time and location of training [32].

Some GPs thought that the quality of the information received from secondary care did impact on their ability to provide care during CCRs [11]: “So it’s the initial diagnosis, that I think generally now the information is excellent, but I think at 6 months or something, often the information isn’t as good, and that was I think what I read quickly, where the patients were wanting their review with us, more formal review.” (practice 2, GP partner, page e180) [11].

This was reflected in Watson’s survey [26] of primary care staff which showed that 29% were satisfied with oncology letters. Primary care staff noted that oncology discharge letters omitted key parts of survivorship care such as familial risk of cancer, psychological and social consequences of cancer and symptoms indicating recurrence. This was reflected in an audit of sixty-five discharge letters in the same study. GPs alluded to a checklist or survivor care plan [11, 26] containing a summary of topics to discuss with GPs as well as previously mentioned topics.

### Outcomes of CCRs

There was only one research paper which identified the effect of 2 CCRs over 6 months, delivered through group consultations with 6 patients at a single GP practice [34]. The group consultations showed a 58% in reduction of physical or emotional concerns and lifestyle needs as assessed by HNAs. The overall level of concern reduced by 36%. Both patients and clinicians reacted positively to group consultation CCRs, but this was not formally assessed.

## Discussion

### Main findings

The ten studies included in this review presented views of different stakeholders such as patients, partners of patients and primary care staff such as GPs through interviews, focus groups and surveys. There was a single study that measured the outcomes of group consultation CCRs using holistic needs assessments, but this outcome had not been previously validated for CCRs both in a group or patient-clinician setting. There were no studies that identified methodology to evaluate CCRs or showed the effect of CCRs on patient quality of life or symptoms. Of those asked, most patients were unable to recall CCRs but those who did recall CCRs did not find them helpful: often favouring a review with their usual GP compared to other GPs. Patients favoured a proactive approach of contact from primary care via CCRs as it legitimised concerns as well as a more comprehensive offering of care beyond the current CCR structure. Both patients and GPs identified barriers such as lack of GP time and poor primary-secondary care coordination. The perception of lack of time to conduct CCRs from patients was reciprocated by policymakers. For some patients, this perception prevented help-seeking behaviour towards healthcare providers. A further barrier for GPs and practice nurses was a lack of knowledge about long term cancer care and relevant resources for signposting. For GPs there was a tension between having a pre-determined but fixed structure relevant to financial incentive to conduct CCRs and providing clinical care which may supersede this structure. There was no consensus on the optimal timing of CCRs. Lastly, partners of patients said that they would value a CCR for their own health and social concerns related to taking on additional roles as a carer which had a physical and emotional burden. Furthermore, a care review for partners would provide time to stay updated with the health of their partner who was living with and beyond cancer and avoid gatekeeping: this needs to be considered in a framework of confidentiality. A synthesis of the main findings, gaps in the research and recommendations for further research can be found in Table 2.

**Table 2:** A summary of the scoping review: clinical practice and research implications using the Pattern, Advances, Gaps, Evidence for Practice and Research recommendations (PAGER) framework from Bradbury-Jones et al. (2021)[24]. There was no evidence of the impact of CCRs on patient symptoms, quality of life or methodology used to evaluate CCRs therefore these were omitted from the table

Pattern	Advances	Gaps	Evidence for practice	Research recommendations
Patient views on CCRs	Few patients recalled having a CCR and those that did remember having a CCR tended to have had a negative experience except when they had a prior relationship with their GP. Some patients felt that GPs did not know enough about their own cancer journey or cancer treatments in general. Other recognised time as a barrier to raising concerns with their primary care teams. A proactive approach to cancer care legitimised concerns but CCRs did not cover many areas such as an explanation of the diagnosis, symptoms of recurrence or details of local support services.	There is a lack of research identifying what leads to positive and negative patient experiences of having CCRs. There has been little research conducted shortly after a CCR when interviews might be less prone to recall bias.	CCRs are limited by a structure which can guide but may dictate care delivery. The current CCR structure may not meet patient cancer care needs.	To carry out qualitative research with patients about their experiences of having CCRs and how their experience could be improved.
Primary care staff views on CCRs	GPs and practice nurses were conflicted by the tick-box nature of CCRs against the usefulness of the prompts in CCR structure. Primary care team members felt constrained by time resources, lack of knowledge in long-term cancer care and inadequate primary-secondary care coordination.	There is a lack of research identifying ways of improving primary care time and personnel resources locally to facilitate CCRs. Furthermore, there is a lack of research on improving primary-secondary care coordination around cancer care at the end of discharge from secondary care.	Primary care staff need time, personnel and educational resources specific to cancer care to adequately deliver CCRs.	To carry out qualitative research with GPs and practice nurses to identify how CCRs are carried out, the impact of the COVID-19 pandemic on CCRs, the role of primary care in long-term cancer care and the perceived impact on patients and their families. To survey and interview secondary care oncology staff about the facilitators and barriers to providing timely documentation to primary care, their ideas about the role of primary care and challenges about communicating with primary care.
Caregiver views on CCRs	Partners of patients wanted to have their own review time beyond the CCR to keep updated with the patient’s health and treatments alongside discussing their own health concerns and being a carer.	There is a lack of research identifying the acceptability and feasibility of interventions to support caregivers: psychological support, psychoeducational interventions and caregiver support both in hospital and community settings.	It is unclear how to deliver care to caregivers especially in the context of CCRs and encouraging a wider approach to support patients and their families would be helpful.	To carry out qualitative research with partners of patients with cancer and wider family members about their supportive care needs. Further research should identify the feasibility for healthcare professionals to support these needs.
Validated outcomes to evaluate CCRs	There are very few validated outcomes that can be used to evaluate CCRs. Holistic needs assessments prior to and after CCRs delivered via 2 group consultations over a 6-month period showed decreased patients unmet needs.	There is a need for research to evaluate the impact of CCRs before and after its implementation both in a patient-practitioner and group consultation setting.	Quality of care delivered in CCRs is rarely measured and there is a need for validated outcomes to evaluate care quality during CCR delivery.	To carry out qualitative research with patients about positive and negative experiences with CCR delivery to inform a validated score which could be used in clinical practice.

### Findings in the context of other studies

Most patients did not recall CCRs possibly because the CCR had not occurred, or that their CCR with a GP or practice nurse was not significant, compared to hospital appointments at the same time. Furthermore, it is possible that the CCRs may have been forgotten due to the stress and burden of cancer treatment as well as cognitive impairment in short-term or working memory related to cancer treatment [35]. It is difficult to determine the impact of CCRs and eliminate recall or attention bias without validated outcome measures.

For nurse-led cancer care assessments or holistic needs assessments (HNAs), survey data from the UK [36, 37], Australia [38] and Canada [37] identified similar barriers to implementation, to those encountered for CCRs, with the biggest barriers being insufficient time and healthcare professionals to carry out assessments. Other barriers included lack of services to which patients could be referred to including those that were culturally sensitive, inadequate professional training and a lack of space to ask intimate questions. Patient-related factors included travelling distance and cost [36]. A systematic review of HNAs [13] found some trials found benefit whilst others found no benefit on quality of life and patient symptoms. Thematic analysis revealed that the way that HNAs were implemented is perhaps more important than the implementation of HNAs themselves: HNAs are a means to an end rather than an end in themselves.

For survivorship care plans (SCPs) in the USA, focus groups [39] and a survey [40] with primary care practitioners identified time and lack of recommendations from oncology teams as barriers to implementation. Lack of knowledge of survivorship issues and lack of survivorship guidance were other barriers to SCP use in clinical practice. Despite this, qualitative data [41] shows that SCPs improve primary care team confidence in managing cancer survivorship sequelae. A meta-analysis of SCPs [15] identified no impact on patient outcomes which is thought to be due to lack of comparability of different SCPs, the inadequate implementation of SCPs or ineffectiveness.

A survey of Canadian primary care staff [42] suggested that a diagnosis and treatment summary was the most useful of all discharge information but US data [43] suggested that less than half of primary care providers surveyed actually received a treatment summary. A systematic review [14] revealed that treatment summaries improved patient perceived care and treatment adherence but had no impact on physical or mental health. The review was limited by observational data and no consistency in outcome reporting making it difficult to draw conclusions.

SCPs are not used in the UK and there are no studies evaluating its use. Whilst treatment summaries are part of the ‘personalised care’ initiative, there are no studies evaluating their use in the UK.

### The role of general practice

A systematic review [44] of the views of patients and GPs on the role of the GP in long-term cancer care, identified that patients often feel abandoned by their hospital cancer teams after treatment and discharge. Many patients were unsure about the exact role of the GP and tended to seek care from the GP only if they had an existing relationship. Both GPs and patients agreed that GPs should provide primary healthcare, act as a care advocate, and contact secondary care to facilitate referrals for example. A separate systematic review [45] focussing on psychosocial care for those living with and beyond cancer identified that patients preferred seeing their GP for depression and anxiety. Alternatively, fear of recurrence was thought to be better managed by oncology teams. Barriers to better cancer care included inadequate communication with secondary care, especially individualised care planning, alongside a lack of survivorship care guidelines and training [44]. There are few cancer care training programs for primary care staff worldwide which show long-term impact on clinician knowledge and patient outcomes [46]. GPs are unsure about their role within cancer care and when to re-establish care after a cancer diagnosis and perhaps CCRs in the first 12 months after a cancer diagnosis do act to re-establish patient contact. Lack of clear guidance is likely to have knock-on effects on patient experience [47]. Patients believed a prominent barrier to GP-delivered cancer care was that GPs were too busy. Perceived time constraints may negatively impact patient help-seeking behaviour and result in unmet care needs [48]. Time and resource scarcity were also echoed as barriers to adequate care provided by GPs in this study. A systematic review [49] comparing primary and secondary care as providers of survivorship care found similar quality of life and patient reported outcomes from several heterogenous randomised controlled trials and observational studies, with up to 5 and 15 years of follow-up respectively. However, primary care was associated with lower costs to patients and society more widely.

### The impact of cancer on the individual and on the wider family

The long-term effects of cancer are wide-ranging from the commonly known physical effects such as fatigue, psychological effects such as depression and social effects such as isolation. Another impact is unemployment directly or indirectly due to cancer and its treatment: ‘financial toxicity’[50]. Notably relationships with family and loved ones more broadly are important for emotional, social and spiritual support [51]. As well as this, the need to provide this support changes the nature of relationships with family members, possibly through a combination of the cancer diagnosis, financial hardship and change in intimate relationships [51, 52]. In a home environment, family caregivers adopt several roles such as symptom monitoring and assessment, care coordination, providing physical support such as giving medications, and psychosocial support [53]. Family caregivers encounter a conflict between being caring duties and their own needs [54] which may explain increased caregiver cardiovascular and psychological morbidity [55] as well as increased all-cause mortality [56]. Family-specific interventions include psychological support, psychoeducation interventions and caregiver support [57].

Psychosocial interventions, namely a provision of psychological and/or social support, directed at caregivers did not have clear short- or long-term changes on caregiver quality of life, physical or psychological health [58]. Psychoeducational interventions which provide an educational component about cancer, in addition to components of psychosocial interventions can result in significant improvements [59] in caregiver physical and psychological health, quality of life and burden at 3 months as well as physical quality of life specifically at up 12 months. Despite this, interventions may be difficult to implement due to limited evaluation of their acceptability, adoption and feasibility [60]. A meta-analysis [61] examining the impact of electronic Health (e-Health) interventions found an improvement in caregiver symptoms and quality of life but not in caregiver burden. The longest of the included 7 randomised control trials (*n* = 326) had a maximum follow-up of 14 weeks [61].

### Conclusions, limitations and future studies

There is currently insufficient evidence to support the implementation of cancer care reviews in clinical practice. Stakeholder views identified barriers to providing CCRs as lack of time, adequate primary-secondary care coordination and lack of knowledge about long-term cancer care and resources for signposting. Patients preferred a proactive care offering such as a CCR, but few could recall if it had taken place. Partners of patients would value a clinical review time for their own health concerns. CCRs can be evaluated by administering HNAs before and after CCRs, and comparing the numbers of physical and emotional concerns, lifestyle needs and total number of concerns. However, evaluation of CCRs using HNAs was only implemented in a group consultation setting.

The survey data included in this review is limited by reporting bias and potentially selection bias as responders were recruited from an online forum [26] or tended to be interested in research participation [28]. Walter and colleagues’ survey [28] contained more male GP participants and fewer part-time trainees than expected. Survey data contained no information about ethnic background [26, 28] or disability. Very few patient views of CCRs were identified and only negative experiences were noted [11]. Patient views may not have been represented in some cases with a lack of people from diverse ethnic backgrounds [27, 30], middle to lower socio-economic classes [30] and those with disabilities. One study with clinicians and policymakers was published 5 years after data collection and 6 of the 10 included studies were published before 2016 and may not be relevant to current practice. This is because there has been significant policy changes such as use of structured templates for CCRs [10] and education for GPs [62] and practice nurses [63]. The most recent 2021–22 changes to CCR policy include a 3-month review soon after diagnosis and a 12-month review after completion of initial treatment which may proactively legitimise concerns which had been absent in previous iterations of CCRs noted in this review [10]. Changes in consulting modalities since the start of the COVID-19 pandemic may mean that included studies may not be relevant to current practice [64].

Further studies should identify the role that primary care practitioners believe they have in CCRs, their perceived usefulness for patients as well as the process for undertaking CCRs (see Table 2). The Care Act 2014 (UK) does highlight the need for primary care providers to support carers, and whilst this is not included formally in CCRs it could be considered for future iterations provided it was accompanied by adequate resourcing [65]. Other work could focus on improving care coordination between secondary care and primary care such as treatment summaries. The views of patients would be valuable to understand the role that CCRs do and should play in delivering long term cancer care. CCRs may very well be improved by telling patients about the role of CCRs and perhaps an electronic or paper printout at the end of the consultation could improve its impact [33]. In addition, further studies on how to support families, specifically caregivers, would be helpful.

## Data Availability

The searches generated during the current study are available from the corresponding author on reasonable request.

## Declarations

### Disclaimer

The views expressed are those of the authors and not necessarily those of the National Health Service (NHS), the NIHR or the Department of Health and Social Care.

## References

[1] Cancer: World Health Organisation; 2022 [cited 2022 08 16]. Available from: https://www.who.int/news-room/fact-sheets/detail/cancer.

[2] Sung H, Ferlay J, Siegel RL, Laversanne M, Soerjomataram I, Jemal A (2021). Global cancer statistics 2020: GLOBOCAN estimates of incidence and mortality worldwide for 36 cancers in 185 countries. CA Cancer J Clin.

[3] Kocarnik JM, Compton K, Dean FE, Fu W, Gaw BL, Collaboration GBoDC (2022). Cancer incidence, mortality, years of life lost, years lived with disability, and disability-adjusted life years for 29 cancer groups from 2010 to 2019: a systematic analysis for the global burden of disease study 2019. JAMA Oncol..

[4] Ahmad AS, Ormiston-Smith N, Sasieni PD (2015). Trends in the lifetime risk of developing cancer in Great Britain: comparison of risk for those born from 1930 to 1960. Br J Cancer.

[5] Routes from diagnosis: Macmillan Cancer Support; 2020 [cited 2021 02 26]. Available from: https://www.macmillan.org.uk/about-us/what-we-do/evidence/using-cancer-data/routes-from-diagnosis.html.

[6] Cancer survival statistics for all cancers combined: Cancer Research UK; [cited 2022 08 16]. Available from: https://www.cancerresearchuk.org/health-professional/cancer-statistics/survival/all-cancers-combined.

[7] Statistics fact sheet: Macmillan Cancer Support; 2021 [cited 2022 08 16]. Available from: https://www.macmillan.org.uk/_images/cancer-statistics-factsheet_tcm9-260514.pdf.

[8] Personalised care and improving quality of life outcomes: NHS 2022 [cited 2022 08 16]. Available from: https://www.england.nhs.uk/cancer/living/.

[9] Holistic Needs Assessments: Macmillan Cancer Support; 2022 [cited 2022 08 16]. Available from: https://www.macmillan.org.uk/healthcare-professionals/innovation-in-cancer-care/holistic-needs-assessment.

[10] Cancer Care Review: Macmillan Cancer Support; 2022 [cited 2022 08 05]. Available from: https://www.macmillan.org.uk/healthcare-professionals/cancer-pathways/prevention-and-diagnosis/cancer-care-review

[11] Adams E, Boulton M, Rose P, Lund S, Richardson A, Wilson S (2011). Views of cancer care reviews in primary care: a qualitative study. Br J Gen Pract.

[12] Quality and Outcomes Framework guidance for 2021/22: NHS England; 2021 [cited 2022 08 05]. Available from: https://www.england.nhs.uk/wp-content/uploads/2021/03/B0456-update-on-quality-outcomes-framework-changes-for-21-22-.pdf.

[13] Johnston L, Young J, Campbell K (2019). The implementation and impact of Holistic Needs Assessments for people affected by cancer: A systematic review and thematic synthesis of the literature. Eur J Cancer Care (Engl).

[14] Corsini N, Neylon K, Tian EJ, Mahpirof E, McLaughlin A, McLeod S (2020). Impact of treatment summaries for cancer survivors: a systematic review. J Cancer Surviv.

[15] Hill RE, Wakefield CE, Cohn RJ, Fardell JE, Brierley M-EE, Kothe E (2020). Survivorship care plans in cancer: a meta-analysis and systematic review of care plan outcomes. Oncologist.

[16] Munn Z, Peters MDJ, Stern C, Tufanaru C, McArthur A, Aromataris E (2018). Systematic review or scoping review? Guidance for authors when choosing between a systematic or scoping review approach. BMC Med Res Methodol.

[17] Arksey H, O’Malley L (2005). Scoping studies: towards a methodological framework. Int J Soc Res Methodol.

[18] Peters MDJ, Marnie C, Tricco AC, Pollock D, Munn Z, Alexander L (2020). Updated methodological guidance for the conduct of scoping reviews. JBI Evid Synth.

[19] Tricco AC, Lillie E, Zarin W, O'Brien KK, Colquhoun H, Levac D (2018). PRISMA extension for scoping reviews (PRISMA-ScR): checklist and explanation. Ann Intern Med.

[20] PRISMA for Scoping Reviews: PRISMA: transparent reporting of systematic reviews and meta-analysesd; 2018 [cited 2022 08 16]. Available from: http://www.prisma-statement.org/Extensions/ScopingReviews.

[21] Berkowitz C, Allen DH, Tenhover J, Zullig LL, Ragsdale J, Fischer JE (2017). Knowledge and preferences of primary care providers in delivering head and neck cancer survivorship care. J Cancer Educ.

[22] Peters MDJ, Godfrey CM, Khalil H, McInerney P, Parker D, Soares CB (2015). Guidance for conducting systematic scoping reviews. Int J Evid Based Healthc.

[23] Survivorship: During and After Treatment: American Cancer Society; 2022 [cited 2022 08 16]. Available from: https://www.cancer.org/treatment/survivorship-during-and-after-treatment.html.

[24] Ouzzani M, Hammady H, Fedorowicz Z, Elmagarmid A (2016). Rayyan-a web and mobile app for systematic reviews. Syst Rev.

[25] Bradbury-Jones C, Aveyard H, Herber OR, Isham L, Taylor J, O’Malley L (2022). Scoping reviews: the PAGER framework for improving the quality of reporting. Int J Soc Res Methodol.

[26] Watson EK, Sugden EM, Rose PW (2010). Views of primary care physicians and oncologists on cancer follow-up initiatives in primary care: an online survey. J Cancer Surviv.

[27] Adams E, Boulton M, Rose PW, Lund S, Richardson A, Wilson S (2012). A qualitative study exploring the experience of the partners of cancer survivors and their views on the role of primary care. Support Care Cancer.

[28] Walter FM, Usher-Smith JA, Yadlapalli S, Watson E (2015). Caring for people living with, and beyond, cancer: an online survey of GPs in England. Br J Gen Pract.

[29] Dilley SP (2019). Can QOF cancer care reviews help with continuity of care?. Br J Gen Pract.

[30] Beaver K, Martin-Hirsch P, Williamson S, Kyrgiou M (2020). Exploring the acceptability and feasibility of patient-initiated follow-up for women treated for stage I endometrial cancer. Eur J Oncol Nurs.

[31] Williamson S, Beaver K, Langton S (2020). Exploring health care professionals views on alternative approaches to cancer follow-up and barriers and facilitators to implementation of a recovery package. Eur J Oncol Nurs.

[32] Dyer S, Dewhurst S (2020). Why general practice nurses need education about cancer as a long-term condition. Primary Health Care.

[33] Kendall M, Mason B, Momen N, Barclay S, Munday D, Lovick R (2013). Proactive cancer care in primary care: a mixed-methods study. Fam Pract.

[34] Hodgson E (2020). Group consultations for cancer care reviews in general practice. Pract Nurs.

[35] Lange M, Joly F, Vardy J, Ahles T, Dubois M, Tron L (2019). Cancer-related cognitive impairment: an update on state of the art, detection, and management strategies in cancer survivors. Ann Oncol.

[36] Wells M, Semple CJ, Lane C (2015). A national survey of healthcare professionals' views on models of follow-up, holistic needs assessment and survivorship care for patients with head and neck cancer. Eur J Cancer Care (Engl).

[37] Williamson S, Hack TF, Bangee M, Benedetto V, Beaver K (2021). The patient needs assessment in cancer care: identifying barriers and facilitators to implementation in the UK and Canada. Support Care Cancer.

[38] Beesley VL, Staneva A, Nehill C, Milch V, Hughes F, Webb PM (2020). Patterns of, and barriers to supportive care needs assessment and provision for Australian women with gynecological cancer and their caregivers: a mixed-methods study of clinical practice. Palliat Support Care.

[39] Hewitt ME, Bamundo A, Day R, Harvey C (2007). Perspectives on post-treatment cancer care: qualitative research with survivors, nurses, and physicians. J Clin Oncol.

[40] Dulko D, Pace CM, Dittus KL, Sprague BL, Pollack LA, Hawkins NA (2013). Barriers and facilitators to implementing cancer survivorship care plans. Oncol Nurs Forum.

[41] LaGrandeur W, Armin J, Howe CL, Ali-Akbarian L (2018). Survivorship care plan outcomes for primary care physicians, cancer survivors, and systems: a scoping review. J Cancer Surviv.

[42] Smith SL, Wai ES, Alexander C, Singh-Carlson S (2011). Caring for survivors of breast cancer: perspective of the primary care physician. Curr Oncol.

[43] Merport A, Lemon SC, Nyambose J, Prout MN (2012). The use of cancer treatment summaries and care plans among Massachusetts physicians. Support Care Cancer.

[44] Meiklejohn JA, Mimery A, Martin JH, Bailie R, Garvey G, Walpole ET (2016). The role of the GP in follow-up cancer care: a systematic literature review. J Cancer Surviv.

[45] Deckx L, Chow KH, Askew D, van Driel ML, Mitchell GK, van den Akker M (2021). Psychosocial care for cancer survivors: a systematic literature review on the role of general practitioners. Psychooncology.

[46] Chan RJ, Agbejule OA, Yates PM, Emery J, Jefford M, Koczwara B, Hart NH, Crichton M, Nekhlyudov L. Outcomes of cancer survivorship education and training for primary care providers: a systematic review. J Cancer Surviv. 2022;16(2):279–302. 10.1007/s11764-021-01018-6 PMC7990618 33763806

[47] Taylor S, Johnson H, Peat S, Booker J, Yorke J (2020). Exploring the experiences of patients, general practitioners and oncologists of prostate cancer follow-up: a qualitative interview study. Eur J Oncol Nurs.

[48] Cromme SK, Whitaker KL, Winstanley K, Renzi C, Smith CF, Wardle J (2016). Worrying about wasting GP time as a barrier to help-seeking: a community-based, qualitative study. Br J Gen Pract.

[49] Vos JAM, Wieldraaijer T, van Weert HCPM, van Asselt KM (2021). Survivorship care for cancer patients in primary versus secondary care: a systematic review. J Cancer Surviv.

[50] Zafar SY, Peppercorn JM, Schrag D, Taylor DH, Goetzinger AM, Zhong X (2013). The financial toxicity of cancer treatment: a pilot study assessing out-of-pocket expenses and the insured cancer patient's experience. Oncologist.

[51] Webb ME, Murray E, Younger ZW, Goodfellow H, Ross J (2021). The supportive care needs of cancer patients: a systematic review. J Cancer Educ.

[52] Essue BM, Iragorri N, Fitzgerald N, de Oliveira C (2020). The psychosocial cost burden of cancer: a systematic literature review. Psychooncology.

[53] Ullgren H, Tsitsi T, Papastavrou E, Charalambous A (2018). How family caregivers of cancer patients manage symptoms at home: a systematic review. Int J Nurs Stud.

[54] Cai Y, Simons A, Toland S, Zhang J, Zheng K (2021). Informal caregivers' quality of life and management strategies following the transformation of their cancer caregiving role: a qualitative systematic review. Int J Nurs Sci.

[55] Möllerberg MLSA, Lithman T, Noreen D, Olsson H, Sjövall K (2016). The effects of a cancer diagnosis on the health of a patient's partner: a population-based registry study of cancer in Sweden. Eur J Cancer Care (Engl).

[56] Nakaya N, Sone T, Tomata Y, Nakaya K, Hoshi M, Shimizu K (2019). All-cause mortality among Japanese whose cohabiting partners are diagnosed with cancer: the Ohsaki Cohort 2006 study. Acta Oncol.

[57] Samuelsson M, Wennick A, Jakobsson J, Bengtsson M (2021). Models of support to family members during the trajectory of cancer: a scoping review. J Clin Nurs.

[58] Treanor CJ, Santin O, Prue G, Coleman H, Cardwell CR, O'Halloran P (2019). Psychosocial interventions for informal caregivers of people living with cancer. Cochrane Database Syst Rev.

[59] Cheng Q, Xu B, Ng MSN, Duan Y, So WKW (2021). Effectiveness of psychoeducational interventions among caregivers of patients with cancer: a systematic review and meta-analysis. Int J Nurs Stud.

[60] Ugalde A, Gaskin CJ, Rankin NM, Schofield P, Boltong A, Aranda S (2019). A systematic review of cancer caregiver interventions: appraising the potential for implementation of evidence into practice. Psychooncology.

[61] Li Y, Li J, Zhang Y, Ding Y, Hu X (2022). The effectiveness of e-Health interventions on caregiver burden, depression, and quality of life in informal caregivers of patients with cancer: a systematic review and meta-analysis of randomized controlled trials. Int J Nurs Stud.

[62] Resources for GPs: Macmillan Cancer Support; 2022 [cited 2022 08 05]. Available from: https://www.macmillan.org.uk/healthcare-professionals/for-your-role/doctor/gp.

[63] Resources for practice nurses: Macmillan Cancer Support; 2022 [cited 2022 08 05]. Available from: https://www.macmillan.org.uk/healthcare-professionals/for-your-role/nurse/practice-nurse.

[64] Murphy M, Scott LJ, Salisbury C, Turner A, Scott A, Denholm R (2021). Implementation of remote consulting in UK primary care following the COVID-19 pandemic: a mixed-methods longitudinal study. Br J Gen Pract.

[65] Marczak J, Fernandez JL, Manthorpe J, Brimblecombe N, Moriarty J, Knapp M (2011). How have the Care Act 2014 ambitions to support carers translated into local practice? Findings from a process evaluation study of local stakeholders' perceptions of Care Act implementation. Health Soc Care Community.

